# Outcomes of Two-Stage Autograft Acetabular Reconstruction for Hip Replacement in Young Patients: A Series of 13 Cases

**DOI:** 10.7759/cureus.79897

**Published:** 2025-03-01

**Authors:** Muhamad Rashidee Ab Rashid, Mohd Yusof Ibrahim, Mohammad Nazir Hassan

**Affiliations:** 1 Orthopedics Department, Hospital Raja Perempuan Zainab II, Kota Bharu, MYS

**Keywords:** acetabular defect reconstruction, arthroplasty, autograft, hip replacement, limb length discrepancy, young patients

## Abstract

Managing complex hip arthritis in young patients poses significant challenges due to the critical need to preserve bone stock for potential future surgical interventions. Two-stage reconstruction can preserve bone, restore limb length, and address acetabular defects to achieve optimal functional outcomes and facilitate future revisions. Structural autografts offer the dual advantages of reduced infection risk and enhanced initial stability. This study evaluates the surgical outcomes of two-stage autograft reconstruction in the management of young patients with acetabular defects. This case series reports our experience using a two-stage structural autograft approach for acetabular defects in total hip replacement. In the first stage, the acetabular defect is reconstructed using a femoral head autograft, followed by a second-stage hip replacement after eight weeks. A total of 13 patients underwent this surgery, with a mean age of 35 years. Patients were followed up for an average of five years. Good press-fit stability was achieved in all cases. Follow-up plain radiographs showed excellent trabecular bridging between the graft and host bone, with no evidence of graft resorption or acetabular cup loosening. All patients demonstrated good functional outcomes, with no complications such as non-union, infection, or aseptic loosening. Only one patient had sciatic nerve palsy which resolved spontaneously. This technique is an excellent option for treating young patients with sizable acetabular defects. It effectively restores bone stock, provides essential biological stability, and facilitates future revision surgery when necessary.

## Introduction

Advanced hip arthritis was clinically diagnosed when the patient presented with debilitating pain, stiffness, and altered gait biomechanics. These symptoms significantly hindered the individual's mobility and ability to maintain gainful employment [1]. The surgical management of advanced hip arthritis in young patients needs a proper plan because it carries significant challenges. Hip-preserving surgical procedures in the context of advanced disease generally do not provide satisfactory pain relief. Additionally, the implications of joint arthroplasty in younger patients present important considerations that warrant careful attention.

The outcomes of total hip replacement (THR) within this demographic can vary widely, mainly due to the diverse array of diagnoses associated with hip disorders, the complexity of the deformities necessitating THR, and the demand for long-term durability. Special attention must be given in performing THR on very young patients, it is fundamental to prioritize thorough preoperative planning, precise implant selection, and comprehensive patient education. Furthermore, employing joint-preservation techniques can facilitate any future hip arthroplasty procedures [2].

In our series, we encountered several complex cases requiring THR that proved to be quite challenging. These cases involved migration of the proximal femur accompanied by limb length discrepancies and an incongruent acetabulum. Our goal is to achieve a stable THR, restore hip biomechanics in the true acetabular position, and ensure sufficient bone stock for potential future surgeries. This technique also addresses limb length discrepancies while minimizing the risk of sciatic nerve injury. The most important is to prevent complications such as implant loosening and infection.

One of the studies stated that the risk of failure using impaction bone grafting and metal mesh is about 13.8% [3]. In another study using structured homologous graft in revision THR, the risk of failure is about 20.5% [4]. Our study aims to assess autograft osteointegration, septic and aseptic loosening in THR for acetabular bone defect in young patients.

## Materials and methods

The technique we describe is suitable for Paprosky II and III acetabular defects with limb length discrepancy. The criteria for THR included severe pain and significant difficulty in walking with disability to perform daily activities. The inclusion criteria are young patient which is clinically indicated for THR with Paprosky II and III acetabular defect. The exclusion criteria are hip in active infection, mentally unstable, and abnormal neurology of the lower limb.

The first stage is reconstructing the acetabular defect with femoral head autograft, followed by the second stage, hip replacement after eight weeks. Preoperatively, all patients were evaluated clinically by the author and their team and proceeded with further assessment. For each patient, complete medical history was obtained and assessed for pain, gait, muscle function, and grade of disability regarding limitation in hip range of motion, including limb length discrepancy.

The total number of patients in this study is 13. The author's team conducted all cases at a single tertiary center. They retrospectively reviewed clinical notes, recording patient demographics, clinical assessment, mobility status, comorbidities, classification of acetabular defects using the Paprosky classification, rehabilitation protocol, and radiographic results. The mean follow-up in this study was five years, with a range of one to nine years.

Surgical technique

**Figure 1:**
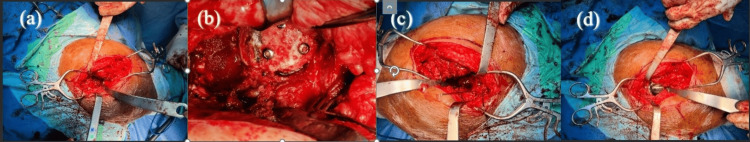
Childhood fracture dislocation right hip and acetabular wall defect complicated with shortening about 5 cm A 39-year-old lady presented with right hip pain, deformity and abnormal gait. First stage surgery (a, b) – reconstruction of the acetabular defect. Exposure of the acetabulum and fixation of the femoral head autograft to acetabular defect by using 3.5mm cortical screw. In this stage, need a lot of fibrosis release for soft tissue traction. It was more challenging when the femoral head was very small for the big defect. In second stage (c, d) – need to assess good incorporation in between graft and acetabulum prior to reaming of the acetabulum and fixation of the acetabulum implant.

**Figure 2:**
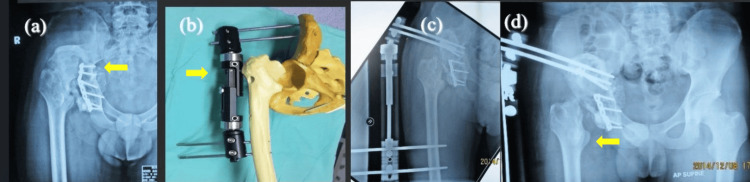
Use of monorail external fixator in case of marked limb length discrepancy > 7 cm These are the x-rays of a 23-year-old male. (a) Preop x-ray: Arrow showed dislocated hip with proximal migration and acetabular defect. (b) In between the first and second stages – an arrow showed using a hip model of monorail application to the iliac bone and femoral shaft for soft tissue distraction. (c) Post op x-ray after application of monorail distractor. (d) X-ray during follow-up showed proximal femur distracted distally (arrow showed level of lesser trochanter more distally compared to before distraction).

In all cases, we use a posterolateral approach. Precise soft tissue was dissected to avoid injury to the sciatic nerve and vascular bundle. The level of the femoral neck was resected and cut at around 1 cm proximal to the lesser trochanter. Soft tissue release and removal of fibrosis together with remnants of abnormal bone (including osteophytes) were done. The true acetabulum was identified before starting reaming. To ensure proper bone incorporation and promote enhanced healing, the acetabulum defect is drilled until healthy bleeding occurs. The defect is then reconstructed using remnants of the femoral head. The cartilage from the remaining femoral head must be removed until the subchondral bone is exposed. The remnant is then reshaped to match the contour of the acetabular defect and stabilized with a 3.5 mm screw, ensuring minimal gap (Figures 1a-1d). The screw must be in proper position for stability and avoid the area of cup placement. For the case of proximal femur migration, which is possible due to soft tissue contraction or dislocations, adequate soft tissue release must be achieved for future surgery (Figures 2a-2d). Intraoperative limb length discrepancy should be less than 3 cm. If more than 3 cm, monorail external fixation (EF) was applied.

In the ward, patients without monorail EF will be applied skin traction for at least two weeks followed by manual intermittent traction at home by family members. Then, patients were allowed to be discharged and taught not to bear weight using crutches. Postoperative patients with monorail EF need to learn the technique of soft tissue lengthening for about 1-2 mm/day, then plan discharge with no weight bearing using crutches. To prevent infection, it is essential to maintain intraoperative sterility. The patient was advised on pin site care, and a prophylactic antibiotic was administered for three days postoperatively. No additional special precautions were included in our technique.

During follow-up appointments in the clinic every three weeks, it is important to assess the condition of the soft tissue to rule out any risk of infection and to evaluate the potential for achieving adequate length for future surgery. Serial x-rays are crucial for assessing the union between the graft and the host bone, and these were performed every three weeks. The second stage of surgery can be done after two months of the reconstruction procedure. The Monorail External Fixator (GPC Medical Ltd., New Delhi, India) must be removed two weeks prior to surgery.

In the second stage of surgery, adhesiolysis and soft tissue release are important to expose the acetabulum and achieve adequate length. The union of graft and host bone must be assessed to prevent graft resorption post-arthroplasty before THR. During primary THR surgery, the structural grafts showed good incorporation and vascularization. Good pressed fit stability was also ensured in all cases. After the surgery, the patient was allowed to use partial weight bearing using crutches.

During follow-up in the clinic, clinical assessment and serial x-ray must be reviewed, at six weeks, three months, six months, one year, and yearly follow-up. This schedule is crucial for assessing trabecular bridging, graft resorption and signs of loosening.

## Results

**Figure 3:**
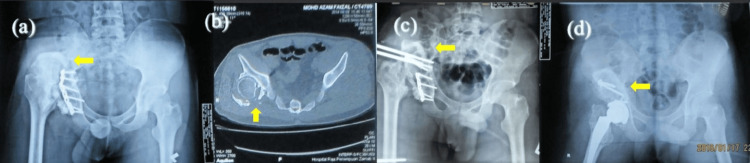
Post acetabular fracture dislocation fixation complicated with acetabular defect, hip stiffness, and limb length discrepancy >7 cm These are the x-rays of a 23-year-old male. (a) Preop x-ray shows migration of proximal femur with osteoarthritis. Arrow showed acetabular defect with false acetabulum. (b) Axial CT scan shows migration proximal femur with acetabular defect (arrow). (c) First stage procedure by application of the monorail and autograft reconstruction of the acetabular defect. Arrow showed a femoral head used to reconstruct the acetabulum. (d) Second stage surgery: removal of monorail and total hip replacement procedure. Interval between first and second stage was 60 days. Arrow showed good osteointegration between the femoral head and the acetabulum defect.

**Figure 4:**
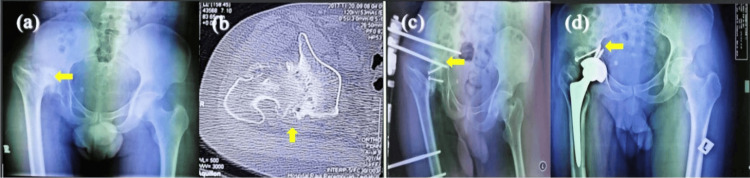
Childhood hip deformity with limb length discrepancy > 7 cm These are the x-rays of a 31-year-old male. (a) Preop: Proximal migration of femur with acetabular defect and pelvic tilted. Arrow showed shallow acetabulum with small femoral head. (b) Axial CT scan shows femoral head deformity with acetabular defect (arrow). (c) First stage procedure by using femoral head autograft for acetabular reconstruction. Arrow showed acetabular defect at superolateral reconstructed use small femoral head. (d) Second stage procedure: Total hip replacement after removal intra acetabular fibrosis. Interval between first and second stage was 60 days. Arrow showed good graft incorporation.

**Figure 5:**
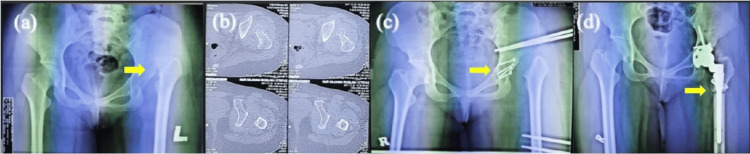
Developmental dysplasia of left hip complicated with limb length discrepancy > 7 cm These are x-rays of a 23-year-old female. (a) Arrow showed Crowe IV DDH with proximal migration of femur. (b) Axial CT scan shows Crowe IV DDH and proximal migration with small femoral head. (c) First stage procedure: reconstruction of acetabular defect with femoral head autograft and monorail application. Arrow showed fixation of the femoral head to the shallow acetabulum. (d) Second stage procedure: acetabulum cup position at true acetabulum during total hip replacement. Interval between first and second stage was 56 days. Arrow showed subtrochantric osteotomy to manage limb length discrepancy.

A total of 13 patients underwent two stage autograft acetabular reconstruction for THR (Table 1). There were four females and nine males with a mean age of 35 years (range 19 to 50 years) with a minimum one-year follow-up (mean, five years; range, one to nine years). First stage involved structural reconstruction of the acetabular defect using femoral head autograft followed by primary THR about six to eight weeks later, for Paprosky type II and III defects. All patients had good functional outcomes and no complications such as non-union, infection and aseptic loosening (Figures 3-5).

**Table 1:** Cases of two-stage procedure in total hip replacement

ID	Gender	Age	Diagnosis	Etiology	Classification (Paprosky/ Crowe)	Interval 1st and 2^nd^ stages	Duration distraction	Surgery date (follow-up duration)	Outcome, Harris Hip Score (HHS)	Complication
1	Male	23	Acetabular fracture with neglected hip dislocation	Road traffic injuries	Paprosky IIIA	60 days	42 days	2014 (9 years)	Shortening >7cm corrected. Good graft incorporation. HHS: Good	No infection and no aseptic loosening
2	Male	58	Posterior wall bone loss with nonunion fracture neck of right femur	Road traffic injuries	Paprosky IIB	56 days	Distractor not applied	2015 (8 years)	Good graft incorporation. HHS: Excellent	No infection and no aseptic loosening
3	Male	25	Left hip avascular necrosis with posterior wall defect	Idiopathic	Paprosky IIB	49 days	Distractor not applied	2016 (7 years)	Good graft incorporation. HHS: Excellent	No infection and no aseptic loosening
4	Male	27	Right femoral head avascular necrosis with superior and posterior wall defect	Idiopathic	Paprosky IIC	49 days	Distractor not applied	2017 (6 years)	Good graft incorporation. HHS: Excellent	No infection and no aseptic loosening
5	Male	31	Neglected slipped capital femoral epiphysis with acetabular defect right hip	Childhood Trauma	Paprosky IIIA	60 days	48 days	2018 (5 years)	Shortening >7 cm corrected. Good graft incorporation. HHS: Good	Sciatic nerve neuropraxia, recovered at 1 year follow up. No infection and no aseptic loosening
6	Female	20	Neglected developmental dysplasia left hip (Crowe 4)	Idiopathic	Paprosky IIIA Crowe IV	56 days	42 days	2018 (4 years)	Shortening >7 cm corrected. Good graft incorporation	No infection and no aseptic loosening
7	Male	27	Neglected closed fracture dislocation left neck of femur with acetabular defect	Road traffic injuries	Paprosky IIB	49 days	Distractor not applied	2019 (3 years)	Good graft incorporation. HHS: Excellent	No infection and no aseptic loosening
8	Female	41	Avascular necrosis of right femoral head with acetabulum wall defect	Idiopathic	Paprosky IIC	56 days	Distractor not applied	2019 (3 years)	Good graft incorporation HHS: Excellent	No infection and no aseptic loosening
9	Male	47	Left hip avascular necrosis post fixation left acetabulum with bone loss	Road traffic injuries	Paprosky IIB	56 days	Distractor not applied	2019 (3 years)	Good graft incorporation. HHS: Excellent	No infection and no aseptic loosening
10	Male	20	Communicated fracture left acetabulum with bone loss and left hip AVN	Road traffic injuries	Paprosky IIB	49 days	Distractor not applied	2019 (3 years)	Good graft incorporation. HHS: Excellent	No infection and no aseptic loosening
11	Female	39	Neglected fracture dislocation right femoral head with acetabular wall defect	Road traffic injuries	Paprosky IIC	49 days	Distractor not applied	2022 (1 year)	Good graft incorporation. HHS: Excellent	No infection and no aseptic loosening
12	Female	46	Left hip dysplasia with osteoarthritis and acetabular wall defect	Idiopathic	Paprosky IIB Crowe IV	56 days	Distractor not applied	2022 (1 year)	Good graft incorporation. HHS: Excellent	No infection and no aseptic loosening
13	Male	50	Post reconstruction acetabular fracture with bone loss and femoral head AVN	Road traffic injuries	Paprosky IIC	58 days	Distractor not applied	2022 (1 year)	Good graft incorporation. HHS: Excellent	No infection and no aseptic loosening

## Discussion

THR in young patients needs proper assessment. Almost all patients who need acetabular reconstruction undergo a standard radiographic evaluation of the pelvis and hip. In general, a computed tomography (CT) scan is an excellent tool for evaluating the loosening of the hardware caused by either mechanical reasons or infection. MRI is most appropriate for evaluating the soft tissues surrounding the hip joint. When patients are evaluated for THR, the surgeon must assess for the amount and type of acetabular bone stock loss.

Management of complex primary THR with acetabular bone loss is almost similar to revision THR. Reconstruction of severe acetabular bone defects during revision total hip arthroplasty (rTHA) represents a challenging procedure with various options including graft, metal augmentation and jumbo cup [5]. Current projections describe a significant upsurge in rTHA of 43%-70% by 2030, especially among patients aged 55-64 and 65-74 years [6]. An appropriate reconstruction method is important to reduce the risk of loosening. This reconstruction method includes the selection of appropriate implants during surgery planning and the precise classification of the bone defect [7]. The correct type of acetabular bone graft is typically selected depending on the classification of the bone stock deficit. While cavitary defects can be repaired using cancellous morselized autografts or allografts, more substantial segmental bone stock defects are typically addressed with structural (or bulk) cortico-cancellous autografts or allografts [8]. First described in 1994, the Paprosky classification was developed to grade acetabular bone loss [9] and has become the most established acetabular defect classification system worldwide [10]. In addition to detailing the severity of bone defects, the classification was also designed to predict the necessary bone grafts and implants. Paprosky types 1 and 2 defects are mostly treated with morselized bone graft, whereas bulk graft is recommended for type 3 defects [9,11]. Several studies have reported good to excellent clinical and radiological outcomes of rTHA using allografts [12-15]. Recent reviews indicate that trabecular metal (TM) systems, which include augments and shells, have shown the best outcomes for reconstructing large acetabular defects, particularly regarding re-revisions and radiographic loosening [5].

Paprosky type II and III acetabular defects for primary THR are challenging. Accurate acetabular reconstruction at the correct level restores biomechanics to nearly normal, utilizing the best available bone stock. The interpretation of adequate bony coverage of the acetabular implant and the use of bone graft to facilitate coverage is a matter of debate. However, most authors agree that if coverage is between 60%-80%, an augmentation procedure (structural graft or medialization of acetabulum or both) should be used. Graft coverage should be at most 30%-40%.

In young and active patients, using structural bone graft to restore bone defects and provide adequate initial support for the revision acetabular component is one such option. However, the main concern is the problem with structural bone graft resorption, which leads to acetabular cup loosening. We have done our two-stage primary THR surgery in a significant acetabular bone defect. In the cases with significant limb length discrepancy, it is more challenging. We are reporting such cases with our technique: using residual femoral head autograft as a structural bone graft to reconstruct the acetabulum, using monorail for soft tissue distraction and bringing down the proximal femur followed by primary THR in the second stage, about eight weeks after the first stage procedure above. As far as we are concerned, our technique has yet to be reported in any journal so far.

In our series, a few advantages are compared with other techniques. We use biological autograft augmentation, which has a lower risk of disease transmission, infection, and immune rejection. The cost of the autograft is significantly cheaper compared to allografts and other augmentation techniques.

In our two-stage surgery, we can use a primary implant that offers greater longevity compared to complex implants. Gradual soft tissue distraction reduces the risk of sciatic nerve injury when compared to acute distraction, especially in cases of marked limb length discrepancy. There is consensus that increasing limb length beyond a certain length is associated with an increased risk of nerve injury. Edwards et al. demonstrated a 28% higher risk of nerve injury in patients with greater than 4 cm lengthening following THA [16]. In our series, we prefer gradual distraction to minimize the risk of nerve injury, particularly in cases of significant limb length discrepancy.

However, every procedure or technique has its disadvantages. In our approach, the risk of infection is slightly higher due to the involvement of two-stage surgeries and the potential for pin tract infections; however, we have not encountered this issue in our cases. The risks associated with anesthesia are also elevated; however, most of our patients are fit for multiple surgeries. The hospitalization costs are higher, and patients may feel inconvenienced due to the need for a two-month gap between the two stages of surgery.

## Conclusions

This technique is one of the best surgical options for young patients with significant acetabular defects. It restores bone stock, provides biological stability and is made more accessible for future revision surgery if required.

This procedure would restore acetabular bone defect, provide primary pressed fit cup stability, ensure vascularization and incorporation of the femoral head autograft bone and thus reduce the risk of bone resorption and loosening. It also gives the patient utmost satisfaction and functional outcome because of the significance of limb length discrepancy settled with a stable, functional hip joint.
